# Real-Time Whole-Genome Sequencing for Surveillance of *Listeria monocytogenes*, France

**DOI:** 10.3201/eid2309.170336

**Published:** 2017-09

**Authors:** Alexandra Moura, Mathieu Tourdjman, Alexandre Leclercq, Estelle Hamelin, Edith Laurent, Nathalie Fredriksen, Dieter Van Cauteren, Hélène Bracq-Dieye, Pierre Thouvenot, Guillaume Vales, Nathalie Tessaud-Rita, Mylène M. Maury, Andreea Alexandru, Alexis Criscuolo, Emmanuel Quevillon, Marie-Pierre Donguy, Vincent Enouf, Henriette de Valk, Sylvain Brisse, Marc Lecuit

**Affiliations:** Institut Pasteur, Paris, France (A. Moura, A. Leclercq, H. Bracq-Dieye, P. Thouvenot, G. Vales, N. Tessaud-Rita, M. Maury, A. Alexandru, A. Criscuolo, E. Quevillon, V. Enouf, S. Brisse, M. Lecuit);; Institut National de la Santé et de la Recherche Médicale Unité 1117, Paris (A. Moura, M.M. Maury, M. Lecuit); Santé Publique France, Saint-Maurice, France (M. Tourdjman, E. Laurent, D. Van Cauteren, H. de Valk);; Ministry of Agriculture, Agrifood, and Forestry, Paris (E. Hamelin, N. Fredriksen, M.-P. Donguy);; Paris Descartes University, Paris (M. Lecuit); Necker-Enfants Malades University Hospital, Paris (M. Lecuit)

**Keywords:** Listeria monocytogenes, bacteria, whole-genome sequencing, WGS, high-throughput DNA sequencing, pulsed-field gel electrophoresis, PFGE, core genome multilocus sequence typing, cgMLST, molecular typing, public health, surveillance, listeriosis, prospective study, France

## Abstract

During 2015–2016, we evaluated the performance of whole-genome sequencing (WGS) as a routine typing tool. Its added value for microbiological and epidemiologic surveillance of listeriosis was compared with that for pulsed-field gel electrophoresis (PFGE), the current standard method. A total of 2,743 *Listeria monocytogenes* isolates collected as part of routine surveillance were characterized in parallel by PFGE and core genome multilocus sequence typing (cgMLST) extracted from WGS. We investigated PFGE and cgMLST clusters containing human isolates. Discrimination of isolates was significantly higher by cgMLST than by PFGE (p<0.001). cgMLST discriminated unrelated isolates that shared identical PFGE profiles and phylogenetically closely related isolates with distinct PFGE profiles. This procedure also refined epidemiologic investigations to include only phylogenetically closely related isolates, improved source identification, and facilitated epidemiologic investigations, enabling identification of more outbreaks at earlier stages. WGS-based typing should replace PFGE as the primary typing method for *L. monocytogenes.*

*Listeria monocytogenes* is a foodborne bacterial pathogen that causes severe illnesses and conditions (*1*) such as septicemia, encephalitis and meningitis, abortion, stillbirths, and neonatal infections (*2*). Although ingestion of *L. monocytogenes* occurs frequently, incidence of listeriosis is generally low (≈6 cases/1 million persons in France) and primarily affects at-risk groups of persons (elderly, those with impaired immunity, pregnant women and their newborns) (*2**,**3*). However, the case-fatality rate for listeriosis is one of the highest among foodborne infections (*2**,**4**,**5*).

On the basis of *L. monocytogenes* typing studies, most listeriosis cases are believed to be sporadic, although numerous listeriosis outbreaks have been reported over the past few decades in Europe and North America (*6**–**10*). In addition to its public health burden (*11*), *L. monocytogenes* can lead to major economic losses in the food industry because of its capacity to replicate at low temperatures and persist on food-processing surfaces despite disinfection (*12**,**13*). Costs associated with recalls of contaminated products (*14*) are high, and international food safety legislations based on microbiological criteria have been established to control *L. monocytogenes* (*15**,**16*). Surveillance programs, including systematic isolate collection and typing, have been established to detect clusters of microbiologically related cases, identify common sources of infection, and take appropriate control measures to reduce human illness and economic losses.

In France, human listeriosis has been a mandatory reportable disease since 1999 (*3*). The French listeriosis surveillance system relies on the National Public Health Agency, which collects epidemiologic data and food consumption histories from all patients with laboratory-confirmed *L. monocytogenes* infection by using a specific hypothesis-generating questionnaire, and the National Reference Centre for *Listeria* (NRCL), which characterizes all human and food isolates received to detect clusters of genetically related strains. Food and environmental investigations are systematically conducted in refrigerators of patients with neurolisteriosis, in hospital kitchens if hospital-acquired *L. monocytogenes* infection is suspected, and among producers of suspected or incriminated products under the authority of the Ministry of Agriculture.

Microbial typing attempts to characterize bacteria at the strain level to detect and investigate clusters of related isolates and identify sources of infection. The standard method for *L. monocytogenes* typing relies on pulsed-field gel electrophoresis (PFGE) and the restriction enzymes *Asc*I and *Apa*I (*17**,**18*). However, the discriminatory power of PFGE is limited compared with whole-genome sequencing (WGS) (*19**–**22*). Core genome multilocus sequence typing (cgMLST) (*23**,**24*) is a highly reproducible method that enables strain comparison across laboratories by using standardized nomenclatures of alleles and types (*20**,**23**–**26*). The power of cgMLST in identifying national or international outbreaks has been demonstrated in several studies and for multiple bacterial species (*10**,**20**,**26**–**30*).

With the advances in sequencing technologies, WGS has become a promising method for routine surveillance to maximize discrimination of isolates. In 2015, the NRCL implemented cgMLST as a typing method for *L. monocytogenes* and has since used it in parallel with PFGE typing. We report the performance of cgMLST as a routine typing tool and its added value for microbiological and epidemiologic surveillance by comparing it with PFGE, the current standard method.

## Materials and Methods

### Bacterial Isolation

The study included 2,743 *L. monocytogenes* isolates prospectively collected during January 1, 2015–December 31, 2016, in the framework of listeriosis surveillance in France. These isolates consisted of 770 from humans, 1,688 from food, and 285 from food production environments. Food and food environmental isolates were obtained from refrigerators or hospital investigations conducted in connection with confirmed human cases, samples from alerts of food companies, and investigations of the Ministry of Agriculture. Pure cultures were obtained by streaking isolated colonies onto Columbia agar plates (bioMérieux, Marcy l’Etoile, France) and incubating overnight at 35°C.

### PFGE Molecular Typing

PFGE profiles were obtained by using restriction enzymes *Asc*I and *Apa*I according to the PulseNet standardized operating procedures (*31*). PFGE runs were performed twice per week, and banding patterns were compared by using the complete linkage clustering algorithm based on number of different bands in BioNumerics version 6.6 (Applied Maths, Sint-Martens-Latem, Belgium); pattern-matching optimization was set at 1%, and band position tolerance was set at 1% (*18*). *Asc*I and *Apa*I profiles were defined for each enzyme separately as differing from other profiles by >2 bands (i.e., a difference of only 1 band per enzyme was tolerated). Combined *Asc*I-*Apa*I PFGE profiles were defined as being of a distinct type for >1 of the 2 enzymes.

### DNA Extraction and WGS

DNA extraction was performed by using the DNeasy Blood and Tissue Extraction Kit (QIAGEN, København Ø, Denmark), from 5 mL of liquid cultures grown overnight at 35°C in brain heart infusion medium under aerobic conditions, following the manufacturer’s protocol for gram-positive bacteria. DNA quantity and purity was assessed by using Qubit fluorimetric quantitation (Thermo Fisher Scientific, Waltham, MA, USA).

Library preparation was conducted by using the Nextera XT DNA Sample Kit (Illumina, San Diego, CA, USA). WGS was performed twice a week on a NextSeq 500 platform (Illumina) by using 2 × 150-bp runs. FqCleaner version 3.0 was used to eliminate adaptor sequences (*32*), reduce redundant or overrepresented reads (*33*), correct sequencing errors (*34*), merge overlapping paired reads (*35*), and discard reads with Phred scores (measure of the quality of identification of nucleobases generated by automated DNA sequencing) <20. Sequences with <40 times average coverage after trimming were resequenced to avoid artifacts in allele calling (*20*). Assemblies were obtained by using CLC Assembly Cell version 4.3.0 (QIAGEN) with estimated library insert sizes ranging from 50 bp to 850 bp and a minimum contig size of 500 bases.

### Sequence-Based Genotyping

cgMLST profiles were extracted from genome assemblies by using the BLASTN algorithm (*36*) implemented in the BIGSdb-*Lm* platform (*20**,**37*), with minimum nucleotide identity of 70%, alignment length coverage of 70%, and word size of 10. Phylogenetic classification based on cgMLST profiles was inferred by using the single linkage algorithm implemented in Bionumerics version 6.6. cgMLST types (CTs) were defined by using international nomenclature (http://bigsdb.pasteur.fr/listeria) based on a cgMLST profile similarity cutoff of 99.600% (i.e., isolates belonging to the same type shared a maximum of 7 allelic differences of 1,748 allele calls) (*20*). cgMLST and PFGE typing results were compared by using the Simpson index of diversity (*38*) and the adjusted Wallace index of concordance (*39*). Rarefaction analyses of richness of a type were conducted by using RStudio version 0.98.485 (RStudio, Inc., Boston, MA, USA).

### Cluster Definition and Epidemiologic Investigations

Before the WGS era, the listeriosis surveillance system in France categorized *L. monocytogenes* strains according to their *Asc*I-*Apa*I PFGE profile frequency in humans. An operational definition of PFGE clusters has been historically set up to identify food sources likely to still be available on the market and thus accessible to appropriate control measures to prevent further cases. A PFGE cluster triggering further epidemiologic investigations (hereby referred to as a PFGE cluster-alert) was defined as >6 human cases during 6 consecutive weeks for endemic PFGE profiles (profiles associated with >12 human cases each year), or as >3 human cases during 6 consecutive weeks for PFGE profiles associated with <12 human cases each year. These different definitions were established to limit time- and resource-consuming investigations, after it was shown that the yield for investigations of PFGE cluster-alerts for endemic profiles did not differ when the >3 human cases threshold was used compared with the >6 human cases threshold during 6 consecutive weeks.

With the implementation of genomic-based surveillance, a pilot definition of cgMLST clusters was set up. We defined a cgMLST cluster triggering further investigations (hereafter referred to as a cgMLST cluster-alert) as a minimum of 2 isolates with the same cgMLST type (CT) identified during 2015–2016, including >1 human isolate.

During the pilot period of 2015–2016, all PFGE and cgMLST cluster-alerts were systematically investigated in parallel. To identify sources of PFGE and cgMLST cluster-alerts, case–case studies were conducted by the National Public Health Agency by using food consumption histories and by comparing food exposures of cluster-related cases with food exposures of sporadic listeriosis cases. Trace-back and trace-forward investigations of suspected or incriminated products were conducted by the Ministry of Agriculture. A food source of infection was considered confirmed if a matching food isolate was recovered from the incriminated food as part of the investigation.

## Results

### Increased Strain Discrimination by cgMLST

**Figure 1:**
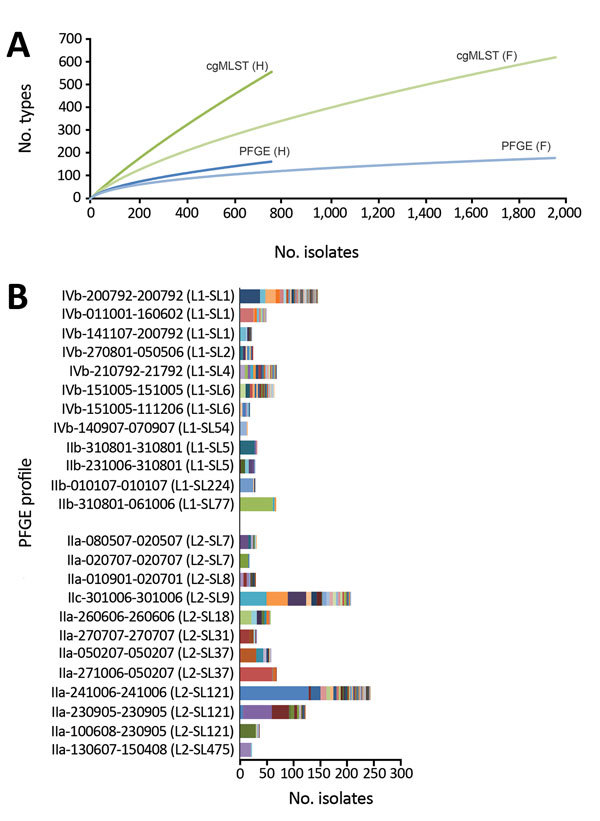
Discriminatory power of pulsed-field gel electrophoresis (PFGE) and core genome multilocus sequence typing (cgMLST) for surveillance of *Listeria monocytogenes*, France. A) Rarefaction analysis of type richness within human (H) and food-associated (F) isolates based on PFGE and cgMLST typing. B) Distribution of number of isolates per PFGE type and cgMLST subtyping. Only the most prevalent PFGE profiles (>20 isolates) are shown. Within each PFGE type, different cgMLST types (CTs) are represented by different arbitrary colors. PFGE types are coded by using the National Reference Centre for *Listeria* internal nomenclature of *AscI*-*Apa*I combined profiles. Information on lineage (L) and sublineage (SL) defined by cgMLST is provided for each PFGE type in parentheses.

Among the 2,743 *L. monocytogenes* isolates prospectively typed during 2015–2016, PFGE identified 268 distinct *Asc*I-*Apa*I combined profiles (Simpson index 0.964, 95% CI 0.962–0.967), whereas cgMLST identified 1,112 CTs (Simpson index 0.992, 95% CI 0.991–0.993) (Figure 1, panel A). Within single PFGE types, 1–280 CTs were identified (Figure 1, panel B). Conversely, 2–7 PFGE types caused by phage insertions and deletions were found in 58 CTs. These results demonstrate that cgMLST significantly increases discrimination of *L. monocytogenes* compared with PFGE (p<0.001 by jack-knife pseudovalues resampling method).

### Increased Number of Cluster-Alerts

**Figure 2:**
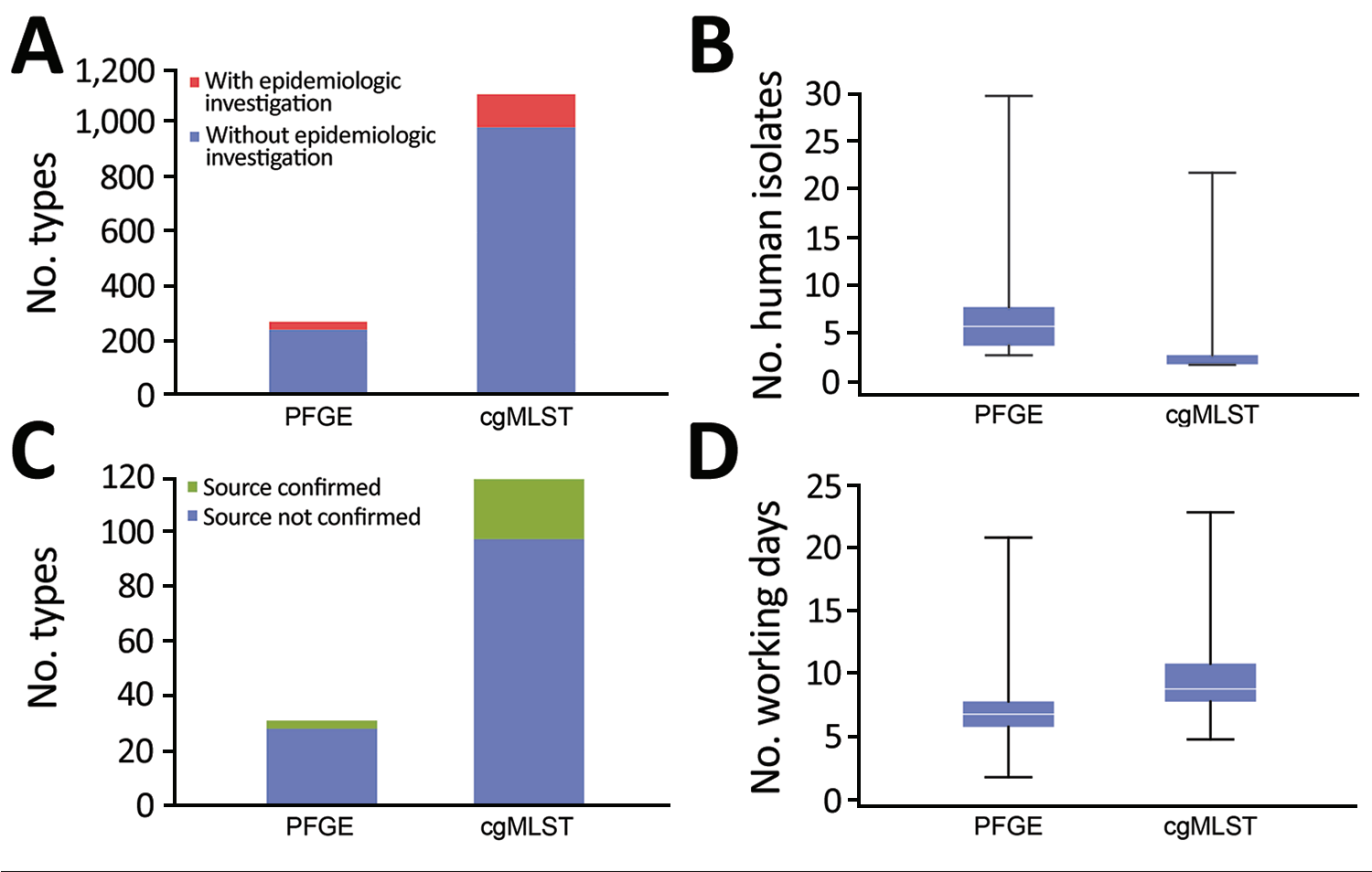
Comparison of pulsed-field gel electrophoresis (PFGE) and core genome multilocus sequence typing (cgMLST) for surveillance of *Listeria monocytogenes*, France. A) Number of total types and number of types triggering epidemiologic alerts. B) Number of human isolates per epidemiologic alert. C) Number of types within epidemiologic alerts with identified source. D) Time delay (days) between obtaining isolate and typing results. Horizontal lines in panels B and D indicate medians, and boxes indicate 25th and 75th percentiles. Error bars indicate maximum and minimum values.

Although the PFGE cluster definition led to identification of 31 PFGE cluster-alerts (Table 1) (245 human isolates), cgMLST cluster definition triggered investigation of 119 cgMLST cluster-alerts (311 human isolates) (Figure 2, panel A). Of these cgMLST cluster-alerts, 37 (31%) involved only 1 human isolate, and 82 (69%) involved >2 human isolates (median 2) (274 human isolates) (Figure 2, panel B) (Table 2). Compared with use of the PFGE cluster definition, we found that use of the cgMLST-based cluster definition resulted in a 3.8-fold increase in cluster-alerts (Figure 2, panel A). The remaining 459 (60%) of 770 human isolates did not match any CTs associated with any other human, food, or environmental isolate identified in 2015–2016 and were considered to be sporadic cases.

**Table 1:** Clusters of PFGE types that triggered epidemiologic investigations for surveillance of *Listeria monocytogenes*, France*

PFGE alert	PFGE type	No. human cases	No. cgMLST types within PFGE alert	Confirmed food source according to PFGE-based investigation
L15/01, L15/06	IIa-260606–260606	7	5	No common source identified, except a specific cheese for 2 cases
L15/02	IVb-210792–210792	8	7	No common source identified, except a specific cheese for 1 case
L15/03	IIa-020707–151007	3	1	NI
L15/04	IIa-010901–020701	3	3	NI
L15/05	IVb-011001–160602	8	8	NI
L15/07	IIb-010107–010107	4	2	Dairy (cheese)
L15/08	IVb-151005–151005	22	18	NI
L15/09	IVb-210792–210792	18	12	No common source identified, except a specific cheese for 4 cases
L15/10	IIb-310801–061006	13	4	Meat (sausage)
L15/11	IVb-011001–160602	17	14	No common source identified, except a specific sausage for 1 case
L15/12	IVb-200792–200792	18	14	NI
L15/13	IVb-270801–050506	6	6	NI
L15/14	IIb-231006–310801	8	5	NI
L15/15	IIa-080507–020507	5	3	NI
L15/16	IIa-010901–020701	6	6	NI
L16/01	IIa-191107–200807	4	1	NI
L16/02	IVb-151005–151005	6	6	NI
L16/03	IVb-151005–111206	4	4	NI
L16/04	IVb-210792–210792	6	5	NI
L16/05	IIa-050207–050207	6	6	NI
L16/06	IVb-270801–050506	3	3	NI
L16/07	IVb-011001–160602	30	11	Dairy (cheese)
L16/08	IIa-260606–260606	3	1	NI
L16/09	IVb-151005–151005	8	8	NI
L16/10	IVb-210792–210792	6	6	NI
L16/11	IIa-271106–271106	4	3	NI
L16/12	IIa-010901–020701	4	4	NI
L16/13	IIa-271106–271106	3	3	NI
L16/14	IVb-210792–210792	7	7	NI
L16/15	IVb-270801–050506	5	5	NI

*cgMLST, core genome multilocus sequence typing; NI, not identified; PFGE, pulsed-field gel electrophoresis.

**Table 2:** Clusters of cgMLST types involving >2 human cases for surveillance of *Listeria monocytogenes*, France*

cgMLST alert	cgMLST type (L-SL-ST-CT)	No. human cases	Minimum cgMLST similarity, %	Confirmed food source according to cgMLST investigation
FR001	L2-SL475-ST504-CT941	9	99.6	NI
FR002	L2-SL14-ST14-CT1265	7	99.8	NI
FR004	L2-SL18-ST18-CT663	2	99.9	NI
FR005	L2-SL18-ST18-CT660	2	99.9	Dairy (raw milk cheese)
FR005a	L2-SL9-ST9-CT1044	2	99.9	NI
FR006	L2-SL16-ST16-CT795	4	100.0	NI
FR007	L2-SL37-ST37-CT572	5	99.9	NI
FR008	L2-SL207-ST207-CT856	2	99.6	NI
FR009	L2-SL71-ST7-CT745	5	99.8	NI
FR009a	L2-SL7-ST7-CT739	2	99.8	NI
FR010	L2-SL451-ST451-CT800	2	99.9	NI
FR011	L1-SL1-ST1-CT1418	4	99.7	NI
FR012	L1-SL1-ST1-CT288	4	99.9	NI
FR013	L1-SL1-ST1-CT300	11	99.9	NI
FR015	L1-SL1-ST1-CT247	2	99.8	NI
FR016	L1-SL1-ST1-CT1394	3	99.9	Dairy (raw goat milk cheese)
FR017	L1-SL1-ST1-CT1401	2	100.0	NI
FR019	L1-SL2-ST2-CT376	2	99.7	NI
FR021	L1-SL6-ST6-CT443	3	99.7	NI
FR022	L1-SL6-ST6-CT1473	4	99.8	NI
FR023	L1-SL6-ST6-CT1465	3	99.9	NI
FR024	L1-SL6-ST6-CT1474	3	99.6	Meat (handmade paté)
FR025	L1-SL6-ST6-CT1475	2	99.8	NI
FR026	L1-SL4-ST4-CT1341	2	99.7	NI
FR027	L1-SL4-ST4-CT168	10	99.9	NI
FR028	L1-SL4-ST4-CT1351	2	99.9	Meat (sausage)
FR030	L1-SL54-ST54-CT1503	6	99.9	Meat (sausage)
FR031	L1-SL77-ST77-CT48	11	99.9	Meat (sausage)
FR032	L1-SL5-ST5-CT1320	2	99.9	NI
FR034	L1-SL5-ST5-CT1331	4	99.9	NI
FR035	L1-SL224-ST224-CT1276	3	99.9	Dairy (raw milk cheese)
FR036	L1-SL54-ST54-CT1501	2	99.8	NI
FR037	L2-SL412-ST412-CT1169	2	99.9	NI
FR038	L2-SL155-ST155-CT1170	3	99.8	NI
FR039	L1-SL1-ST1-CT1424	3	100.0	NI
FR041	L1-SL5-ST5-CT1325	6	99.9	NI
FR045	L1-SL4-ST4-CT165	2	99.7	NI
FR047	L2-SL7-ST7-CT1138	5	99.8	NI
FR051	L2-SL9-ST9-CT594	2	99.7	NI
FR053	L2-SL8-ST8-CT1147	2	99.9	NI
FR054	L1-SL5-ST5-CT1326	2	100.0	NI
FR055	L2-SL403-ST403-CT1107	3	100.0	NI
FR057	L1-SL77-ST77-CT49	7	100.0	NI
FR058	L2-SL7-ST624-CT1142	5	99.8	NI
FR059	L1-SL6-ST6-CT1460	2	99.7	Dairy (raw milk cheese)
FR061	L1-SL6-ST6-CT1486	3	99.9	NI
FR062	L1-SL4-ST4-CT1363	3	99.8	NI
FR066	L2-SL20-ST20-CT1081	2	99.9	NI
FR067	L1-SL2-ST2-CT1533	2	99.7	NI
FR068	L2-SL8-ST8-CT1151	2	99.6	NI
FR069	L1-SL1-ST1-CT1415	2	99.9	NI
FR070	L1-SL59-ST59-CT1281	2	99.8	Dairy (raw milk cheese)
FR071	L2-SL37-ST37-CT1604	3	99.6	NI
FR073	L1-SL1-ST1-CT2060	2	99.9	NI
FR075	L1-SL59-ST59-CT1728	3	99.9	NI
FR076	L1-SL5-ST5-CT1308	2	99.7	NI
FR079	L2-SL18-ST18-CT1420	4	99.8	NI
FR081	L1-SL6-ST6-CT1977	2	99.8	NI
FR082	L2-SL200-ST200-CT1641	2	99.9	Meat (dried sausage)
FR083	L1-SL1-ST1-CT2056	22	99.7	Dairy (cheese)
FR084	L1-SL87-ST87-CT1294	2	99.9	NI
FR085	L1-SL6-ST6-CT1488	2	99.9	NI
FR086	L2-SL8-ST8-CT1154	2	99.7	NI
FR087	L2-SL7-ST7-CT1863	2	99.7	Dairy (raw cow milk cheese)
FR088	L2-SL415-ST394-CT1111	2	99.8	NI
FR091	L2-SL155-ST155-CT1171	3	99.7	NI
FR096	L1-SL1-ST1-CT2072	2	99.9	NI
FR097	L1-SL4-ST4-CT1357	2	100.0	NI
FR098	L1-SL4-ST4-CT1339	2	99.8	NI
FR099	L2-SL8-ST8-CT1929	2	99.9	NI
FR104	L1-SL1-ST1-CT1982	2	99.9	NI
FR105	L2-SL101-ST101-CT2118	2	99.9	NI
FR106	L1-SL1-ST1-CT1381	2	99.9	NI
FR107	L1-SL1-ST1-CT1396	2	99.9	NI
FR108	L1-SL4-ST408-CT1365	2	99.6	NI
FR109	L1-SL87-ST87-CT1743	4	99.7	NI
FR111	L2-SL14-ST14-CT958	2	99.9	NI
FR119	L1-SL220-ST220-CT1432	2	99.8	NI
FR121	L2-SL101-ST101-CT1526	2	99.9	NI
FR122	L1-SL1-ST1-CT2055	2	99.7	NI
FR123	L1-SL87-ST87-CT1293	2	99.7	NI
FR124	L1-SL5-ST5-CT1987	2	99.9	NI

*cgMLST, core genome multilocus sequence typing. NI, not identified.

Inherent to the different criteria used to define cgMLST and PFGE cluster-alerts, cgMLST cluster-alerts contained lower numbers of human isolates compared with PFGE cluster-alerts (median 2 isolates in cgMLST vs. median 6 in PFGE cluster-alerts) (Figure 2, panel B). Median time from isolate reception to typing results was 7 working days for PFGE and 9 working days for WGS (Figure 2, panel D).

### Facilitation of Detection of Food Sources

Epidemiologic investigations identified a confirmed food source in 3 (10%) of 31 PFGE cluster-alerts (Table 1). A confirmed food source was identified in 22 (18%) of 119 cgMLST cluster-alerts (Figure 2, panel C; Table 3).

**Table 3:** Source identification of cgMLST cluster-alerts for surveillance of *Listeria monocytogenes*, France*

cgMLST cluster-alert size, no. human isolates	No. (%)				
	Clusters, n = 119	Human isolates, n = 770	Food/environment isolates, n = 1,973	Clusters with confirmed source	Cluster-alerts with identified source resulting in withdrawal or recall
Small, 1	37 (31)	37 (5)	145 (7)	10 (27)	3 (30)
Medium, 2–5	73 (61)	185 (24)	123 (6)	9 (12)	5 (55)
Large, >5	9 (8)	89 (12)	83 (4)	3 (38)	0

*cgMLST, core genome multilocus sequence typing.

Among the 37 cgMLST cluster-alerts involving only 1 human isolate, a cgMLST-matching food source of infection was identified in 10 (27%) (Table 3). All of these sources were identified during investigations of refrigerators of patients with neurolisteriosis and after confirmed exposure to the incriminated product. In 9 of 10 of these investigations, the food source was also identified by PFGE, and food strains matched corresponding profiles of human isolates. In 3 (30%) of these 10 conclusive investigations, a product withdrawal and recall was issued as a direct consequence of the investigation (Table 3) and probably contributed to preventing further infections. Among the remaining 27 cgMLST cluster-alerts involving only 1 human isolate and >1 food/environmental isolate (Table 3), although these isolate(s) provided an immediate hypothesis, no exposure to the food item from which the matching strain was isolated could be confirmed.

Among the 82 cgMLST cluster-alerts involving >2 human isolates (Table 3), a confirmed source of infection was identified for 12 (15%): in 3 (6%) of 53 cgMLST cluster-alerts that contained only human isolates, and in 9 (31%) of 29 cgMLST cluster-alerts containing >1 food/environmental isolate. For cgMLST cluster-alerts containing >1 food/environmental isolate, matching food/environmental isolate(s) immediately provided a strong hypothesis and facilitated identification of the food source. In 5 (42%) of these 12 conclusive investigations, a product withdrawal and recall was issued as a direct consequence of the investigation and probably contributed to preventing additional human infections (Table 3). No product withdrawal or recall was issued as a direct consequence of investigations of the 31 PFGE clusters-alerts.

Among 70 (85%) of 82 cgMLST cluster-alerts involving >2 human isolates with no source of infection identified, a 6-month follow-up period after identification of the cluster-alert was available for 59 (84%). After identification of these 59 unsolved cgMLST clusters-alerts with 6-month follow-up, no additional cgMLST-matching human case was identified in 43 (73%) of 59, making identification of a source of infection unlikely to have prevented a major number of cases. These clusters without further development might have resulted from point-source contamination of short shelf-life food products, rather than long-term infection of the food-production environment. In 5 (8%) of these 59 unsolved cgMLST clusters-alerts, additional cgMLST-matching human cases occurred within 3 months of cluster identification and none was identified afterwards, suggesting that early identification of a food source would have likely prevented further listeriosis cases. Finally, in 11 (19%) of the 59 unidentified cgMLST clusters-alerts involving >2 human isolates, additional cgMLST-matching human cases occurred >6 months after cluster identification. These clusters could potentially be linked to persistent *L. monocytogenes* contamination of food production environments. Strengthening capacities to identify sources of such clusters is likely to prevent further illnesses by identifying safety gaps in food production plants.

### False PFGE Cluster-Alerts

Despite the usefulness of PFGE in identifying clusters of listeriosis cases over the past few decades, the limited discriminatory power of PFGE can indicate that unrelated isolates are indistinguishable, thus leading to identifying and investigating false PFGE cluster-alerts. The increased discriminatory power of cgMLST enabled identification of such false cluster-alerts. In 2015, PFGE cluster-alert L15/08 (Table 1) consisted of 22 human isolates with indistinguishable *Asc*I-*Apa*I combined PFGE profiles (IVb-151005–151005) among nationally distributed cases with onsets spanning >5 months. Despite intensive and time-consuming investigations, including iterative case–case studies as new cases were reported, no common source was identified. cgMLST showed that this PFGE cluster-alert did not consist of highly related isolates: 18/22 isolates had distinct CTs, and 3 cgMLST cluster-alerts (FR022, FR023, and FR025) (Table 2) could be distinguished within this PFGE cluster-alert. Investigations of these cgMLST cluster-alerts were inconclusive, but using cgMLST clustering rather than PFGE clustering would have saved public health resources at national and local levels.

### Detection of Clusters Not Detected by PFGE

For 12 (15%) of 82 cgMLST cluster-alerts involving >2 human cases, human isolates exhibited >1 PFGE profile (i.e., corresponded to clusters of highly related isolates that were undetected by PFGE). In cgMLST cluster-alert FR013 (L1-SL1-ST1-CT300) (Table 2), 11 nationally distributed human cases with onsets spanning >12 months were identified and only 4 of them were part of a detected PFGE cluster-alert. Investigation of this cgMLST cluster-alert resulted in several potential sources of infection, none of which could be confirmed because of incomplete trace-back information.

### Better Identification of Outbreak-Associated Cases

In 2015, cgMLST cluster-alert FR028 (L1-SL4-ST4-CT1351) (Table 2) identified 2 human isolates matching 1 food isolate recovered from producer A, which was sampled as part of a food alert of the Ministry of Agriculture. None of the case-patients reported having consumed that specific food. Concomitantly, cgMLST cluster-alert FR030 (L1-SL54-ST54-CT1530) (Table 2) identified 6 human isolates in the same geographic area that matched several deli meat items, including a sausage type, from producer A. Food and environmental investigations showed polyclonal contamination of the food production environment of this producer, and a case–case study conducted for all cases implicated in these 2 clusters showed an association with consumption of sausage. Products from producer A were withdrawn and recalled, and no further human cases were identified afterward. Because onsets of the 6 human cases involved in cgMLST cluster-alert FR030 were outside the 6-week period required to define a PFGE cluster-alert, this outbreak was not identified by PFGE.

### Detection of Long-Term Persistent *L. monocytogenes* Strains

In June 2015, an investigation conducted at a local producer patronized by a patient in whom neurolisteriosis subsequently developed showed food and environmental infections with a distinct CT as the clinical strain. Intensive cleaning and disinfection was implemented at this production facility. In September 2016, investigation of another human case of neurolisteriosis in the same geographic area showed that the case-patient patronized a farmers’ market where food from the previously implicated producer was sold. Investigations at the producer’s facility identified persistent contamination with the same CT as the 2015 isolates (FR043, L2-SL121-ST121-CT914). Because of the 15-month interval, this persistent contamination would have been missed by the PFGE-based cluster definition.

### Identification of Outbreaks after Regulatory Food Testing

In May 2016, an investigation was conducted on a specific cheese type from a major cheese producer in France and identified low-level (<10 CFU/g) *L. monocytogenes* contamination of the final product. The firm issued a nationwide withdrawal of 30 tons of cheese, but no recall was issued according to national regulatory criteria. Food isolates were sequenced at the NRCL and did not have the same CT as any human isolate at that time. In the following 3 months, consistent with the 3-month shelf-life of the implicated product, 22 human isolates had the same CT (L1-SL1-ST1-CT2056; FR083 cluster-alert) (Table 2) and were identified nationwide. Case–case analysis confirmed a significant association between illness and consumption of the implicated product (p = 0.00001). Subsequent food testing confirmed that the implicated cheese was the source for this outbreak. During the same period, PFGE identified 30 human isolates that matched food strains (IVb-011001–160602). However, with cgMLST typing, the analysis was restricted to isolates that were closely related genetically, not just to those that were of the same PFGE profile. This analysis enabled the statistical association to be defined more precisely. This retrospective outbreak was the largest identified in France since 2000.

## Discussion

Because listeriosis is a rare infection and primarily affects at-risk populations, listeriosis outbreaks tend to be small and difficult to control. As in most countries, PFGE-based *L. monocytogenes* molecular subtyping has been used for molecular surveillance of this bacterium in France. Since 1999, PFGE identified multiple outbreaks, which led to major improvements in food safety. However, WGS has shown the limited discrimination and occasional lack of accuracy of PFGE (*19**,**20**,**40*). PFGE does not reflect phylogeny, and it lacks the resolution to distinguish bands of nearly identical sizes. For certain profiles that are highly prevalent, these limitations can result in insufficient discrimination and can hinder appropriate detection of clusters.

We showed the usefulness and feasibility of WGS for prospective *L. monocytogenes* surveillance by using cgMLST and demonstrated its added value over PFGE. cgMLST detected clusters not detected by PGFE, which enabled elimination of several pseudoclusters defined by PFGE and detection of closely related isolates with different PFGE profiles caused by phage insertions or deletions. It also identified clusters of cases associated with persistent food production environmental contamination that were previously unrecognized.

cgMLST results were obtained within 9 working days, compared with 7 working days for PFGE. This difference showed that use of cgMLST for real-time surveillance is feasible because these intervals can be shortened by improved laboratory processing and technological advances.

The unprecedented discriminatory power of cgMLST has provided an opportunity to revisit the criteria used in France since 1999 to define clusters of isolates triggering further investigations. Instead of 3–6 PFGE-matching human isolates identified within a defined period, clusters triggering investigation are now defined by a minimum of 2 genetically related isolates, including >1 human isolate regardless of the time interval. However, this procedure has created novel challenges for public health and regulatory agencies to formally prove an outbreak food source through epidemiologic investigations. With more clusters of smaller size being identified by cgMLST, traditional analytic epidemiology is challenged because of the lack of statistical power, and identification of a food source for these small clusters tends to more extensively rely on trace-back or trace-forward investigations, rather than on case–case studies. Increasing demands of product trace-back or trace-forward investigations should be expected as a consequence of increasing identification of small-size clusters by cgMLST. Timely results from these investigations will be fundamental to initiate control measures in instances where a lower level of epidemiologic evidence is available and will likely contribute to an increased number of reported outbreaks (i.e., investigated cluster-alerts with an identified source of infection).

Implementation of WGS-based surveillance has shown that most clusters involving >2 human isolates with no source of infection identified did not progress over time. This finding is consistent with the observation that most listeriosis case clusters in France are linked to local products that have limited production and distribution. 

With the increasing identification of small size clusters by cgMLST, we envision that centralized collection of human, food, and environmental bacterial genomes, including those from regulatory food testing, into dedicated genomic databases (e.g., BIGSdb-Lm and GenomeTrackr) will enable detection of unusual sources of infection and identify atypical vehicles for contamination. The ability to promptly document cases’ food consumption histories and to constantly adapt hypothesis-generating questionnaires to newly identified vehicles will be crucial to improve investigations and identify more outbreaks, enabling better control and measures to be implemented. 

In conclusion, implementation of WGS for routine preventive L. monocytogenes surveillance increases discrimination of isolates, leading to detection of more clusters of related isolates at an earlier stage than PFGE. Optimization of costs and delays is a challenge, which is strongly balanced by gains in genotypic precision. Public health and regulatory agencies will need to adapt their investigation methods to novel challenges raised by WGS-based surveillance. These challenges might lead to better strategies to control L. monocytogenes in food-processing plants, and ultimately help reduce the risk for infection. On the basis of results of this prospective study, PFGE typing has been discontinued, and L. monocytogenes surveillance in France has relied on cgMLST since January 2017.
