# Correction: Vol. 23, No. 9

**DOI:** 10.3201/eid2311.C22311

**Published:** 2017-11

**Keywords:** errata, erratum, correction

A link to Table 2 online was incorrect in the print and PDF versions of Real-Time Whole-Genome Sequencing for Surveillance of *Listeria monocytogenes*, France (A. Moura et al.). The PDF of the article has been corrected online (https://wwwnc.cdc.gov/eid/article/23/9/17-0336_article).

